# Circulating MicroRNAs as potential biomarkers for cerebral collateral circulation in symptomatic carotid stenosis

**DOI:** 10.3389/fphys.2024.1403598

**Published:** 2024-11-01

**Authors:** Wenwen Liang, Bingcang Huang, Qin Shi, Xuelian Yang, Hanwen Zhang, Wei Chen

**Affiliations:** ^1^ Department of Radiology, Gongli Hospital of Shanghai Pudong New Area, Shanghai, China; ^2^ Department of General Practice, Gongli Hospital of Shanghai Pudong New Area, Shanghai, China; ^3^ Department of Neurology, Gongli Hospital of Shanghai Pudong New Area, Shanghai, China

**Keywords:** symptomatic carotid stenosis, cerebral collateral circulation, MicroRNAs, biomarkers, potential biomarkers

## Abstract

**Background:**

Cerebral collateral circulation (CCC) considerably improves the prognosis of patients with symptomatic carotid stenosis (SCS). This study evaluated the diagnostic value of plasma microRNAs (miRNAs) in determining CCC status in patients with SCS.

**Methods:**

This single-center observational study enrolled patients with ≥50% carotid artery stenosis diagnosed using Doppler ultrasound. CCC was assessed using cerebrovascular digital subtraction angiography (DSA). Quantitative reverse transcription–polymerase chain reaction was used to determine the expression levels of plasma miRNAs. A multivariate logistic regression model and receiver operating characteristic (ROC) curve were used to analyze the diagnostic value of plasma miRNA expression in determining CCC status.

**Results:**

A total of 43 patients were enrolled (28 with CCC and 15 without CCC). The plasma expression levels of miR-126-3p, miR-132-3p, and miR-210-3p were significantly higher and those of miR-16-3p and miR-92-3p were significantly lower in patients with CCC. After adjusting for age, gender, drinking history, comorbidities and degree of SCS, miR-92a-3p, miR-126-3p, miR-132-3p, and miR-210-3p were found to be significantly associated with CCC establishment (*p* < 0.05). ROC curve analysis indicated a high diagnostic value of these miRNAs in determining CCC status [area under the curve (AUC): 0.918–0.965], with miR-126-3p exhibiting the highest predictive performance (AUC: 0.965). Subgroup analysis revealed that patients with CCC who had 50%–70% stenosis showed significantly higher expression level of miR-126-3p, whereas those with CCC who had 70%–99% stenosis showed significantly higher expression levels of miR-126-3p, miR-132-3p, and miR-210-3p as well as significantly lower expression levels of miR-15a-3p, miR-16-3p, and miR-92a-3p.

**Conclusion:**

The results indicate that these six plasma miRNAs have promising diagnostic value in determining CCC status in varying degrees of SCS. These miRNAs can serve as biomarkers for CCC status following SCS, with miR-126-3p showing the strongest positive correlation.

## 1 Introduction

Carotid stenosis, the narrowing of extracranial carotid arteries due to atherosclerosis that particularly affects internal carotid arteries or both common and internal carotid arteries, is a significant risk factor for ischemic stroke (Heck and Jost, 2021). Symptomatic carotid stenosis (SCS) notably increases the risk of ischemic events, including transient ischemic attacks, retinal artery occlusion, amaurosis fugax, and stroke, occurring within a period of 6 months (Abbott et al., 2015). This spectrum of atherosclerotic conditions ranges from intima-media thickening to severe stenosis, potentially leading to stroke (Bonati et al., 2022). Hence, timely and effective clinical interventions are crucial.

Cerebral collateral circulation (CCC) comprises a network of blood vessels that maintain cerebral blood flow in cases of primary route failure (Faizy et al., 2022). Recent studies have indicated the role of collaterals in stroke pathophysiology, highlighting their influence on arterial recanalization, reperfusion, hemorrhagic transformation, and neurological outcomes post-stroke (Uniken Venema et al., 2022). Currently, CCC assessment primarily relies on costly or invasive imaging techniques (Liu et al., 2018a). The status of CCC varies among individuals and is influenced by genetic and modifiable risk factors (Maguida and Shuaib, 2023). Therefore, identifying a biomarker for CCC status may have significant clinical importance.

MicroRNAs (miRNAs) are small noncoding RNAs, approximately 22 nucleotides in length (Ho et al., 2022). They act by suppressing translation or inducing the degradation of downstream mRNA targets, thereby modulating gene expression at the post-transcriptional level (Rani and Sengar, 2022). Studies have demonstrated that miRNAs play a crucial role in angiogenesis and vascular remodeling (Çakmak and Demir, 2020; Zhao et al., 2020). Indeed, three classes of miRNAs could be dissociated: pro-angiogenic miRNAs, anti-angiogenic miRNAs and miRNAs with a dual role (Sun et al., 2023). Among several miRNAs, miR-126 (Chen et al., 2017), miR-210 (Raju et al., 2020), and miR-132 (Anand et al., 2010) can promote angiogenesis, whereas miR-92 (Loyer et al., 2014) and the miR-15/16 family (Spinetti et al., 2013; Yin et al., 2012) inhibit it. miR-126, strongly expressed by endothelial cells (ECs), has been identified as a pro-angiogenic factor, which acts by decreasing SPRED-1 level and stimulating the Erk1/2 signaling pathway (Nammian et al., 2020). Previous reports have shown that elevated values of plasma miRNA-126–3p are a prognostic marker for both major adverse cardiovascular events (MACE), as well as for a combined atherosclerosis-driven disease such are coronary artery disease (CAD) and stroke, independent of albuminuria (Martinez-Arroyo et al., 2023). miR-210 has a good potential to promote angiogenesis. Overexpression of miR-210 in mice model of myocardial infarction (MI) promotes peri-infarct revascularization, with a significant upregulation of VEGF expression and a mild increase in HIF-1α expression (Hui et al., 2020). The proangiogenic effect of miR-132 was associated with downregulation of the expression level of the target gene RASA1 in human umbilical vein ECs (HUVECs), which was an important negative regulator of vascular sprouting and vascular branching (Xu et al., 2021). Endothelium-targeted deletion of miR-15a/16-1 not only promoted post-stroke angiogenesis by enhancing the expression of vascular endothelial growth factor A (VEGFA), fibroblast growth factor 2 (FGF2), vascular endothelial growth factor receptor 2 (VEGFR2), and fibroblast growth factor receptor 1 (FGFR1), but also ameliorates post-ischemic blood-brain barrier dysfunction via up-regulating claudin-5 expression (Yang et al., 2017). miR-92a is an endogenous repressor of the angiogenic program in ECs and downregulation of miR-92a is associated with decreased levels of CD93, which is a pro-angiogenic molecule (Piani et al., 2023).

Bin et al. (2016) reported that early-stage acute cerebral infarction can be assessed using the peripheral blood levels of these miRNAs, which are indicative of the CCC status. miR-126, miR-132, and miR-210, in particular, are associated with the development of collateral circulation in acute cerebral infarction.

However, the role of miRNAs in SCS-induced CCC remains unclear, and their levels in peripheral blood have not yet been investigated as biomarkers for CCC status in patients with SCS. In our study, we screened patients with SCS who had ≥50% stenosis using Doppler ultrasound, measured peripheral blood miRNA levels via polymerase chain reaction (PCR), assessed CCC formation through digital subtraction angiography (DSA), and performed multifactor statistical analysis to explore the relationship between SCS and CCC. Our goal was to investigate the association between these biomarkers and CCC in patients with SCS.

## 2 Materials and methods

### 2.1 Study population

**FIGURE 1:**
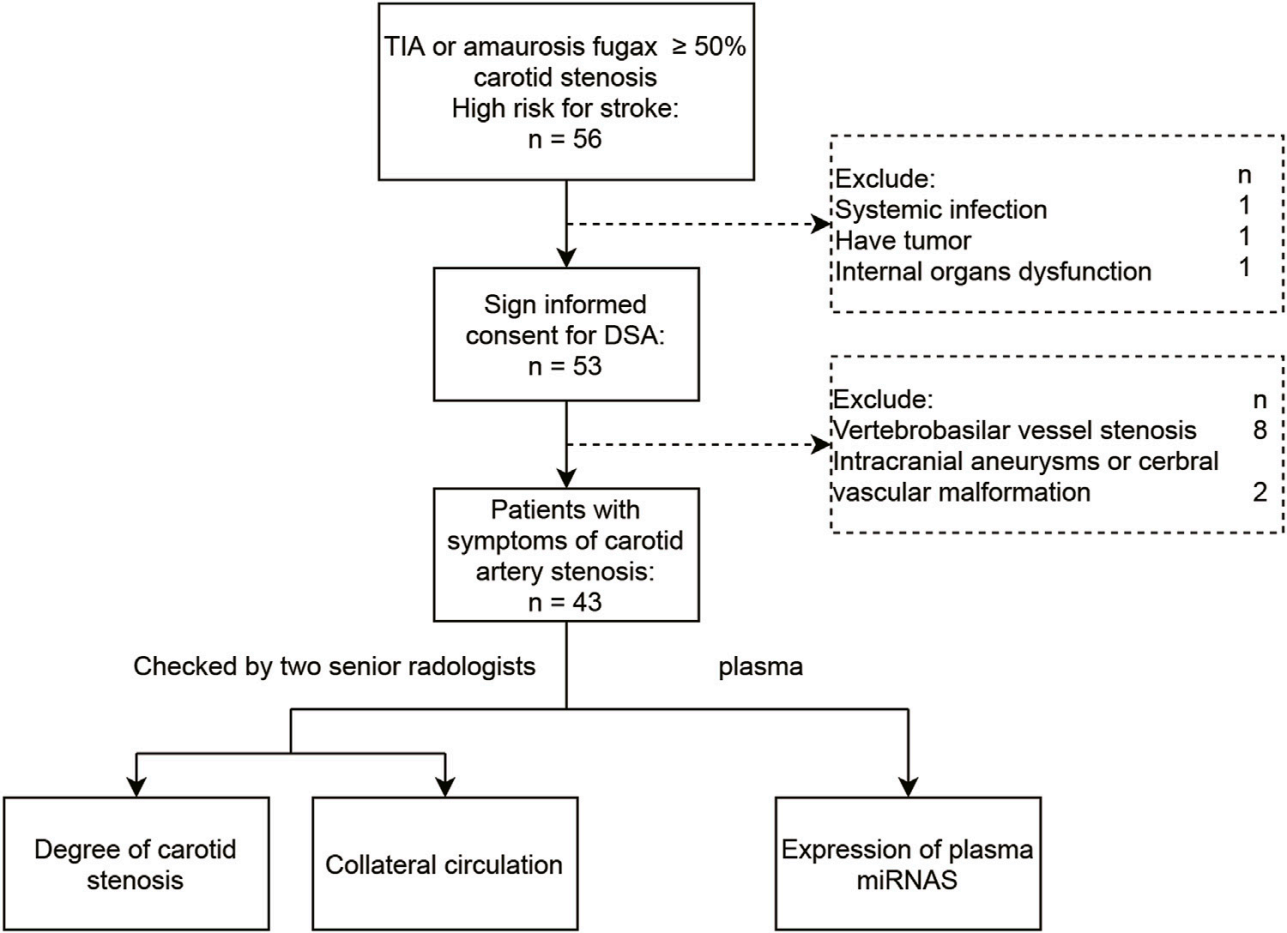
Flow chart of patient selection in the current study.

All patients who presented to the comprehensive stroke center at Shanghai Gongli Hospital with SCS-related concerns between January 2017 and October 2018 were screened for inclusion in this study. This was a single-center, cross-sectional, retrospective observational study by a team of vascular neurologists. SCS usually refers to a series of clinical manifestations caused by insufficient blood supply to the brain as a result of carotid artery stenosis to a certain degree, such as transient ischemic attack (TIA) or amaurosis fugax. The patient selection process is illustrated in Figure 1. Briefly, patients were initially diagnosed with moderate or severe carotid stenosis using a clinical EPIQ7 ultrasound system coupled to a clinical L12-3 transducer (Philips Healthcare, Andover, MA, United States), and the diagnosis was confirmed via computerized angiography (CTA; Siemens, Erlangen, Germany) or magnetic resonance angiography (MRA; Canon, Tokyo, Japan). The stenosis was located at the bifurcation of the common carotid artery and origin of the internal carotid artery, involving a stenotic carotid artery (≥50%) on noninvasive imaging (CTA or MRA). Furthermore, multiple atherosclerotic lesions of the internal carotid artery system (anterior circulation vessels) may be involved. To accurately evaluate the degree of vascular stenosis and intracranial collateral circulation, DSA was performed after obtaining the consent of the patients and their families.

The inclusion criteria of this study were as follows: 1) patients aged ≥18 years; 2) those hospitalized in the neurological department who had undergone cerebral angiography at Shanghai Gongli Hospital; and 3) those with moderate or severe stenosis (≥50%) of the carotid artery, presenting with ischemic events such as transient ischemic attacks, retinal artery occlusion, amaurosis fugax, and stroke.

The exclusion criteria included the presence of vertebrobasilar artery (posterior circulation system) stenosis, intracranial vascular malformations and aneurysms, and brain tumors. Patients with systemic infections, tumors, autoimmune diseases, and severe liver and kidney dysfunction were also excluded.

The demographic, radiographic, and clinical characteristics of all patients were obtained retrospectively. Speciflcally, the data collected included demographics (age and sex), risk factors of stroke (diabetes, heart disease, hypertension, hyperlipidemia, hyperuricemia, smoking, and alcohol consumption), stroke characteristics (previous small infarcts), and radiological findings (carotid stenosis grade and collateral circulation status [open or not]). Hypertension was defined as a systolic blood pressure level of ≥140 mmHg and/or diastolic blood pressure level of ≥90 mmHg. Hyperlipidemia was defined as serum total cholesterol level of ≥5.17 mmol/L (200 mg/dL) and/or serum triglyceride level of ≥1.69 mmol/L (150 mg/dL). Hyperuricemia has been defined as a fasting serum urate concentration >420 μmol/L in men and >360 μmol/L in women (Shan et al., 2021). Additionally, smoking history was defined as cumulative smoking of >100 cigarettes to date. Drinking history was defined as drinking more than once weekly, with the amount of drinking being more than two standard amounts for men and more than one standard amount for women. A standard amount refers to alcoholic beverages containing 12 g of ethanol.

This study was approved by the Ethics Committee of Shanghai Gongli Hospital. Written informed consent was obtained from all patients prior to carotid intervention in accordance with the requirements of the institutional ethics committee.

### 2.2 Cerebrovascular DSA

DSA can accurately detect morphological changes in the arterial wall and is useful for the evaluation of cerebral circulation. The patients underwent cerebrovascular DSA. DSA is helpful in evaluating the characteristics of carotid stenosis (e.g., stenosis site and degree of stenosis); determining the integrity of the contralateral carotid artery, vertebral artery, and intracranial circle of Willis; and assessing whether there is collateral circulation. The femoral artery was punctured using a modified Seldinger technique, and aortic arch angiography was performed using a 5-F pigtail catheter. The openings of major vessels in the bow were observed. The common carotid and subclavian arteries were then selected for cerebral angiography using a 5-F single-curve catheter. Vascular images in the arteriovenous phase were fully displayed at a rate of four frames per second. Angiography was performed in both positive and lateral directions of the common carotid artery stenosis.

The grade of carotid artery stenosis was evaluated according to the North American Symptomatic Carotid Endarterectomy Trial (NASCET) criteria (NASCET et al., 1991). The patients enrolled in this study had a carotid artery diameter reduction of ≥50%. The patients were categorized into two groups according to the degree of stenosis. (Bonati et al., 2021): 50%–70% stenosis (nonsevere stenosis group, NSSG) and 70%–99% stenosis (severe stenosis group, SSG).

CCC status was defined at three levels based on the 2017 Chinese Expert Consensus on Evaluation and Intervention of Collateral Circulation in Ischemic Stroke (Liu et al., 2018b): primary collateral circulation indicates the compensation of blood flow through the circle of Willis, which is the most critical method of compensation; secondary collateral circulation indicates the compensation of blood flow between the ophthalmic artery, pia meningeal anastomosis branch, and other relatively small collateral and collateral anastomosis branches; and tertiary collateral circulation emerges with neovascularization, which is formed after a period of ischemia. The angiography results of all patients were assessed independently by two senior radiologists to confirm the establishment of CCC.

### 2.3 Quantitative reverse transcription (qRT)–PCR miRNA assay

Venous blood samples were collected from each patient on the day of DSA angiography and transferred to a laboratory within 2 h after collection while being maintained at 4°C. Plasma samples were stored at −80°C until serological testing. Total RNA from each plasma sample was extracted using TRIzol LS reagent (Ambion; Thermo Fisher Scientific, Massachusetts, United States), and qRT–PCR was performed using a TaqMan MicroRNA Reverse Transcription Kit (Applied Biosystems; Thermo Fisher Scientific, Massachusetts, United States) (Svec et al., 2015). The expression levels of miR-92-3p, miR-15a-3p, miR-16-3p, miR-126-3p, miR-210-3p, and miR-132-3p were measured using qRT–PCR; U6 was selected as the internal reference. The following primers were used: miR-92-3p forward: 5′-AAG​CGA​CCT​ATT​GCA​CTT​GTC​C-3′; miR-15a-3p forward: 5′-AAC​ACG​CCA​GGC​CAT​ATT​GTG-3′; miR-16-3p forward: 5′-AAT​CGG​CGC​CAG​TAT​TAA​CTG​T-3′; miR-126-3p forward: 5′-AAC​ACG​CTC​GTA​CCG​TGA​GTA-3′; miR-210-3p forward: 5′-AAC​AAG​CTG​TGC​GTG​TGA​CA-3′; miR-132-3p forward: 5′-AAG​CGC​CTT​AAC​AGT​CTA​CAG​C-3′; U6 forward: 5′-CTC​GCT​TCG​GCA​GCA​CA-3′. The relative expression levels of miRNAs were determined using the comparative threshold cycle (2^−ΔΔCT^) method. Each patient’s sample was tested at least three times.

### 2.4 Statistical analyses

Clinical characteristics were summarized and analyzed using descriptive statistics. Continuous variables were expressed as means, whereas categorical variables were expressed as proportions. Differences between groups were evaluated using the chi-squared (χ^2^) test for categorical data and *t*-test for continuous variables. Multivariate analysis was performed using a logistic regression model. Risk factors (e.g., sex, smoking, drinking, and age) were used to adjust odds ratios (ORs) and 95% confidence intervals. Receiver operating characteristic (ROC) curves were used to evaluate the predictive power of circulating miRNAs for determining the CCC status of patients. The area under the curve (AUC) was used to assess predictive power. All statistical tests were two-tailed, with statistical significance set at *p* < 0.05. All analyses were conducted using Statistical Package for the Social Sciences version 25 (IBM Corp., Armonk, NY, United States).

## 3 Results

### 3.1 Characteristics of the study population

Of the 53 patients who originally signed the consent form and were included in the study, 10 were excluded (i.e., eight diagnoses of vertebrobasilar vessel stenosis and two diagnoses of intracranial aneurysm or cerebral vascular malformation after inclusion). Therefore, 43 patients with symptomatic carotid artery stenosis of ≥50% were included in the study (Figure 1). Table 1 shows the clinical information and medical history of patients who had SCS with or without CCC. The study population comprised 26 men and 17 women with a mean age of 66.53 (age range: 33–83) years. The history of drinking was significantly different between patients with and without collateral circulation (*p* < 0.05). The degree of stenosis was significantly different between patients with and without collateral circulation (*p* < 0.05). There were no significant differences in other demographic and clinical characteristics, such as age, gender, obesity [defined as BMI ≥28 kg/m2 according to the Chinese WGOC definition (Zhou and Cooperative Meta-Analysis Group of the Working Group on Obesity in China, 2002)], history of heart disease, hypertension, hyperuricemia, and smoking history, between the two groups (Table 1).

**TABLE 1:** Demographic and clinical characteristics of patients with or without collateral circulation.

Clinical features	Total (N = 43)	CCC		P
		No (N = 15)	Yes (N = 28)	
Gender, n (%)				
Male	26 (60.5)	10 (66.7)	16 (57.1)	0.778
Female	17 (39.5)	5 (33.3)	12 (42.9)	
Age (mean ± SD)	66.53 ± 7.59	67.16 ± 7.58	65.82 ± 7.68	0.502
Body-mass index (BMI), n (%)				
<28	34 (79.9)	12 (80.0)	22 (78.6)	0.999
≥28	9 (20.1)	3 (20.0)	6 (21.4)	
Smoking history, n (%)	25 (58.1)	8 (53.3)	17 (60.7)	0.741
Drinking history, n (%)	9 (20.9)	1 (6.7)	8 (28.6)	0.017
Small infarct, n (%)	19 (44.2)	5 (33.3)	14 (50.0)	0.086
Heart disease, n (%)	9 (20.9)	5 (33.3)	4 (14.3)	0.672
Hypertension, n (%)	38 (88.4)	12 (80.0)	26 (92.9)	0.218
Hyperlipidemia, n (%)	9 (20.9)	3 (20.0)	6 (21.4)	0.999
Hyperuricemia, n (%)	12 (27.9)	4 (26.7)	8 (27.6)	0.999
Diabetes, n (%)	13 (30.2)	3 (20.0)	10 (35.7)	0.534
Degree of carotid stenosis, n (%)				
50%–70% (NSSG)	21 (48.8)	11 (73.3)	10 (35.7)	0.027
>70% (SSG)	22 (51.2)	4 (26.7)	18 (64.3)	

### 3.2 Plasma miRNA levels were correlated with SCS and CCC

**FIGURE 2:**
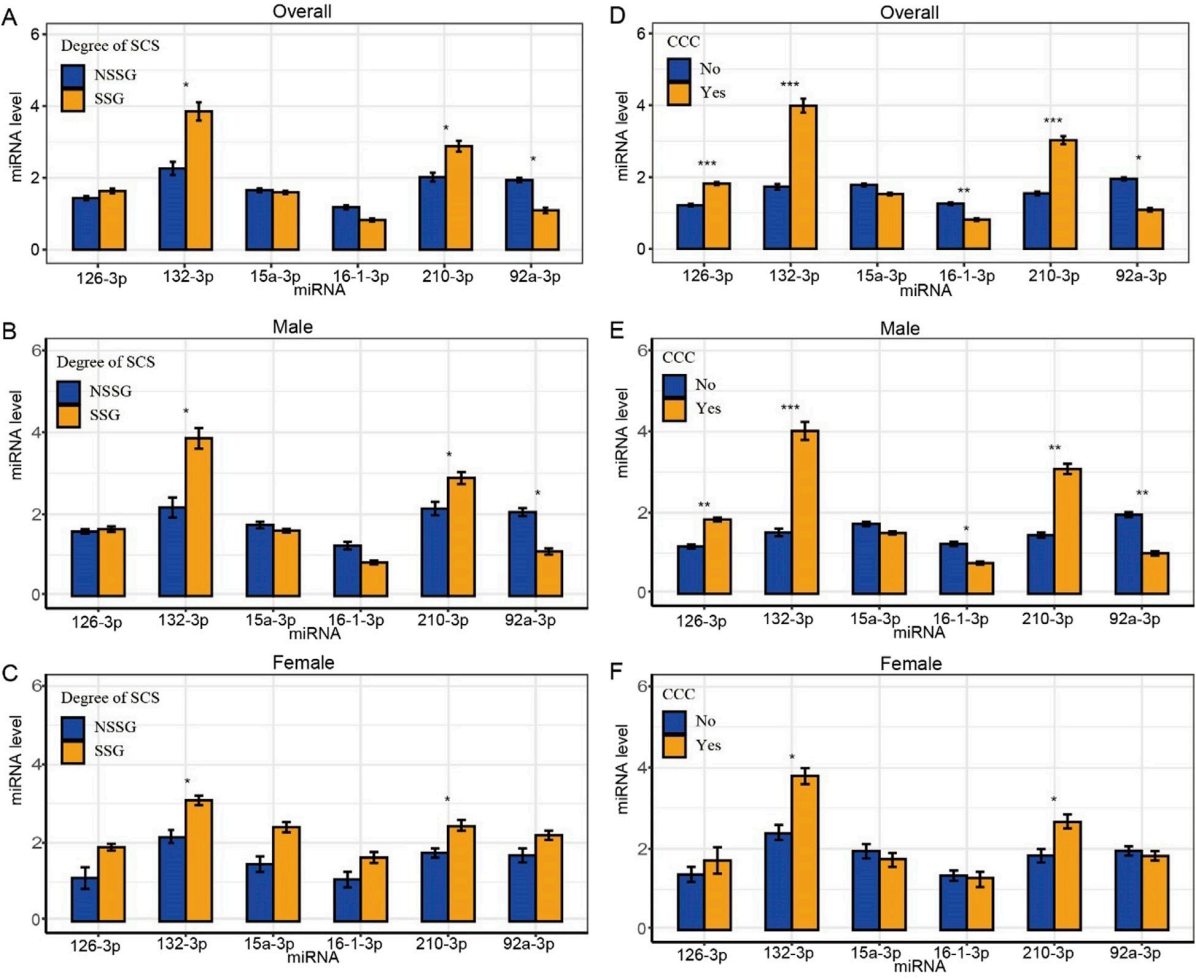
Differences in plasma miRNA levels in patients with different degrees of SCS and CCC. Note: *statistical test *p*-value < 0.05, **statistical test *p*-value <0.01, ***statistical test *p*-value <0.001. **(A)** Differences in plasma miRNA levels in patients subgrouped by degrees of stenosis; **(B)** Differences in plasma miRNA levels in male patients subgrouped by degree of stenosis; **(C)** Differences in plasma miRNA levels in female patients subgrouped by degree of stenosis; **(D)** Differences in plasma miRNA levels in patients subgrouped by CCC; **(E)** Differences in plasma miRNA levels in male patients subgrouped by CCC; **(F)** Differences in plasma miRNA levels in female patients subgrouped by CCC.

We analyzed plasma miRNA expression levels in different SCS and CCC groups. Compared with the NSSG (50%–70% stenosis), the SSG (70%–99% stenosis) showed lower expression levels of miR-92a-3p and higher expression levels of miR-132-3p and miR-210-3p (*p* < 0.05) (Figure 2A). The expression levels of plasma miR-16-3p and miR-92a-3p were lower in patients with CCC than in those without CCC. In contrast, miR-126-3p, miR-132-3p, and miR-210-3p showed higher expression levels in patients with CCC than in those without CCC (*p* < 0.001) (Figure 2D).

Furthermore, we analyzed plasma miRNA expression levels in male and female patients across different SCS and CCC groups separately. In the male subgroup, compared to the NSSG group (50%–70% stenosis), the SSG group (70%–99% stenosis) exhibited lower expression levels of miR-92a-3p and higher expression levels of miR-132-3p and miR-210-3p (*P* < 0.05) (Figure 2B). Additionally, plasma miR-16-3p and miR-92a-3p levels were lower in patients with CCC compared to those without CCC, whereas miR-126-3p, miR-132-3p, and miR-210-3p were elevated in patients with CCC (Figure 2E). In the female subgroup, the SSG group (70%–99% stenosis) showed higher expression levels of miR-132-3p and miR-210-3p compared to the NSSG group (50%–70% stenosis) (*P* < 0.05) (Figure 2C). Similarly, patients with CCC exhibited higher plasma levels of miR-132-3p and miR-210-3p compared to those without CCC (*P* < 0.05) (Table 2 and Figure 2F).

**TABLE 2:** Differences in plasma miRNA levels in patients subgrouped by CCC and degrees of stenosis.

miRNA	CCC within NSSG		CCC within SSG		P_a_	P_b_
	No (N = 11)	Yes (N = 10)	No (N = 4)	Yes (N = 18)		
miR-15a-3p	1.71 ± 0.55	1.59 ± 0.69	2.03 ± 0.60	1.50 ± 0.43	0.681	0.048
miR-16-1-3p	1.34 ± 0.62	0.99 ± 0.66	1.26 ± 0.43	0.73 ± 0.45	0.243	0.041
miR-92a-3p	2.13 ± 0.66	1.70 ± 0.65	2.48 ± 0.58	0.78 ± 0.52	0.16	<0.001
miR-126-3p	1.13 ± 0.48	1.80 ± 0.51	0.79 ± 0.59	1.82 ± 0.64	0.008	0.008
miR-132-3p	1.53 ± 0.52	3.16 ± 2.57	1.40 ± 0.65	4.40 ± 2.77	0.053	0.047
miR-210-3p	1.53 ± 0.62	2.62 ± 1.64	1.30 ± 0.74	3.23 ± 1.55	0.057	0.026

P_a_ represents the p-value of the logistic regression analysis in patients with or without CCC in the NSSG. P_b_ represents the p-value of the logistic regression analysis in patients with or without CCC in the SSG.

### 3.3 Independent diagnostic value of miRNA

**FIGURE 3:**
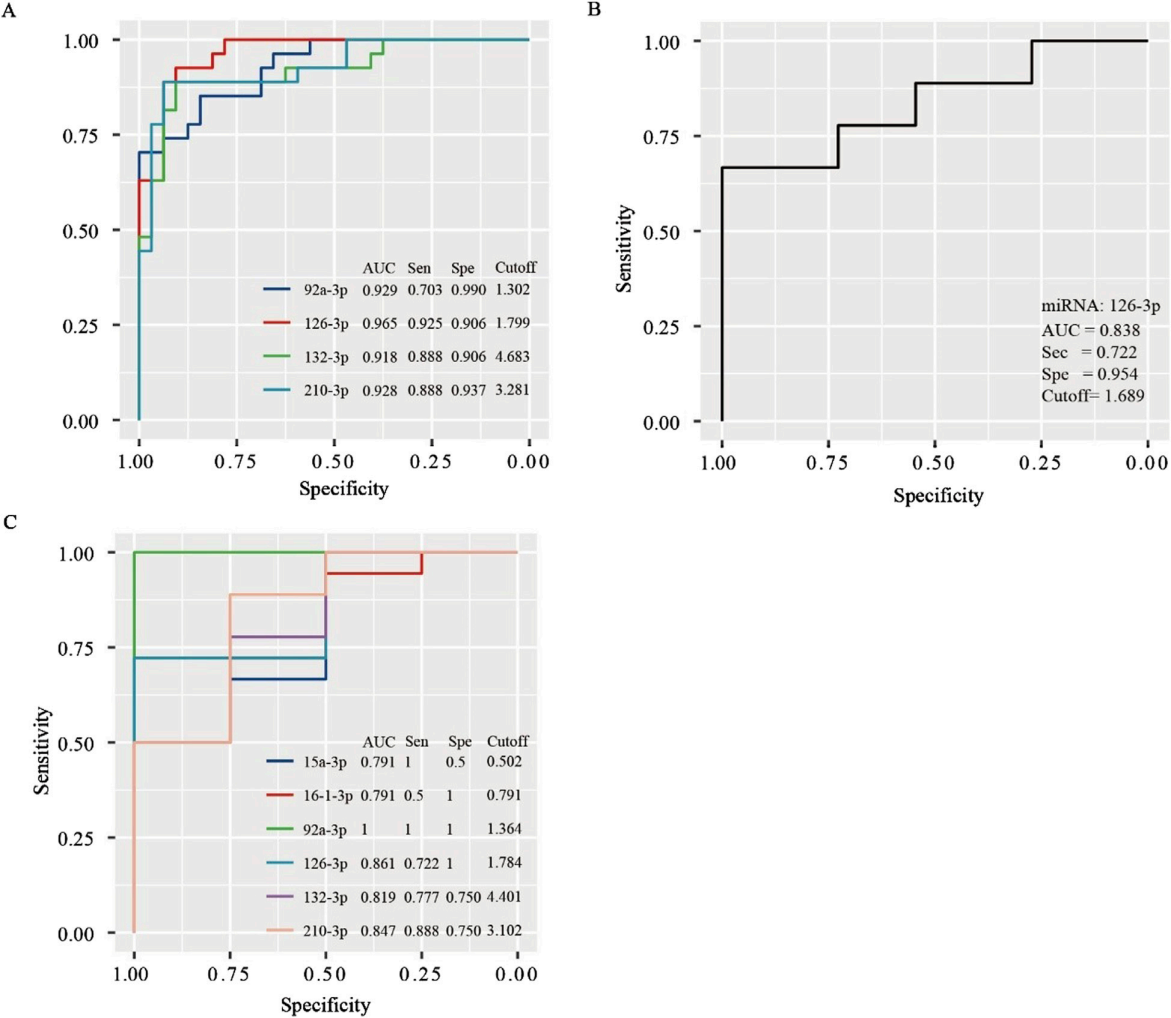
ROC curves for determining the diagnostic value of miRNAs in predicting collateral circulation status in the entire population **(A)**, non-SCS group **(B)**, and SCS group **(C)**.

After adjusting for age, gender, comorbidities, drinking history and the degree of SCS, logistic regression analysis identified miR-92a-3p, miR-126-3p, miR-132-3p, and miR-210-3p as significant factors in determining the CCC status post-SCS. Notably, miR-92a-3p exhibited a negative correlation with CCC establishment (OR = 0.21), whereas miR-126-3p (OR = 44.31), miR-132-3p (OR = 1.91), and miR-210-3p (OR = 3.09) demonstrated positive correlations (Table 3). ROC curve analysis indicated that miR-92a-3p, miR-126-3p, miR-132-3p, and miR-210-3p possess high predictive power for determining CCC status (Figure 3A). In the male subgroup, compared to the NSSG group (50%–70% stenosis), the SSG group (70%–99% stenosis) exhibited lower expression levels of miR-92a-3p and higher expression levels of miR-132-3p and miR-210-3p (*P* < 0.05) (Figure 2B). Additionally, plasma miR-16-3p and miR-92a-3p levels were lower in patients with CCC compared to those without CCC, whereas miR-126-3p, miR-132-3p, and miR-210-3p were elevated in patients with CCC (Figure 2E). In the female subgroup, the SSG group (70%–99% stenosis) showed higher expression levels of miR-132-3p and miR-210-3p compared to the NSSG group (50%–70% stenosis) (*P* < 0.05) (Figure 2C). Similarly, patients with CCC exhibited higher plasma levels of miR-132-3p and miR-210-3p compared to those without CCC (*P* < 0.05) (Figure 2F).

**TABLE 3:** Logistic regression results of miRNAs as biomarkers for CCC status after adjusting for age, gender, drinking history, comorbidities (hypertension, hyperlipidemia, hyperuricemia, and diabetes), and degree of SCS.

miRNA	P value	OR	OR 95%CI
miR-15a-3p	0.404	0.53	0.11–2.35
miR-16-3p	0.178	0.37	0.08–1.47
miR-92a-3p	0.017	0.20	0.04–0.65
miR-126-3p	0.013	54.05	13.96–2.86e + 3
miR-132-3p	0.031	2.60	1.33–8.46
miR-210-3p	0.047	3.74	2.48–12.68

### 3.4 Subgroup analysis

We further performed a subgroup analysis of patients in the NSSG and SSG. In the NSSG, no significant difference was observed in the expression levels of miR-15a-3p, miR-16-3p, miR-92a-3p, miR-132-3p, and miR-210-3p between patients with and without CCC. However, in this group, patients with CCC showed higher expression level of miR-126-3p than those without CCC (*p* < 0.01). Figure 3B shows the ROC curve of miR-126-3p in NSSG patients. When the cutoff value was 1.689, the AUC of miR-126-3p was 0.838, and the sensitivity and specificity were 72.20% and 95.4%, respectively, indicating that miR-126-3p had good diagnostic accuracy for CCC in the NSSG group.

In the SSG, significant differences were observed in the expression levels of miR-15a-3p, miR-16-3p, miR-92a-3p, miR-126-3p, miR-132-3p, and miR-210-3p between patients with and without CCC. Notably, the expression levels of miR-92a-3p, miR-15a-3p, and miR-16-3p were higher in patients without CCC (*p* < 0.05 for miR-15a-3p and miR-16-3p; *p* < 0.01 for miR-92a-3p). Conversely, the expression levels of miR-126-3p, miR-132-3p, and miR-210-3p were higher in patients with CCC (*p* < 0.05 for miR-132-3p and miR-210-3p; p < 0.01 for miR-126-3p). ROC curve analysis indicated the high diagnostic value of these miRNAs in determining CCC status in patients with SCS (Figure 3C).

## 4 Discussion

This study revealed that the incidence of CCC increases with increasing degree of stenosis. miR-15a-3p, miR-16-3p, miR-92a-3p, miR-126-3p, miR-132-3p, and miR-210-3p were all influencing factors of CCC status following SCS. miR-15a-3p, miR-16-3p, and miR-92a-3p were negatively correlated with CCC establishment, whereas miR-126-3p, miR-132-3p, and miR-210-3p were positively correlated with CCC establishment. ROC curve analysis suggested that miR-92a-3p and miR-126-3p have a higher predictive power for determining CCC status in patients with SCS.

SCS is closely associated with ischemic stroke. In the NASCET study, patients with >70% stenosis had a stroke rate of 24% after 18 months, and those with 50%–69% stenosis had a stroke rate of 22% over 5 years (Anand et al., 2010). The incidence of stroke varies greatly among patients with SCS. In clinical practice, all patients with severe carotid artery stenoses do not ultimately develop stroke. The formation of CCC/neovascularization is an important compensatory mechanism for improving cerebral blood perfusion and reducing the occurrence of ischemia (Loyer et al., 2014). In general, a carotid artery stenosis rate of >70% can lead to CCC/neovascularization (Spinetti et al., 2013). In this study, collateral vessel formation was observed in patients with >50% carotid artery stenosis, and CCC occurred in most patients with >70% carotid artery stenosis.

We demonstrated that miR-92a-3p, miR-126-3p, miR-132-3p, and miR-210-3p were all influencing factors of CCC status post-SCS development after adjusting for confounding factors, such as drinking history and degree of carotid stenosis. miR-92a-3p was negatively correlated with CCC establishment, whereas miR-126-3p, miR-132-3p, and miR-210-3p were positively correlated with CCC establishment. ROC curve analysis suggested that miR-92a-3p, miR-126-3p, miR-132-3p, and miR-210-3p had higher predictive power for determining CCC status in patients with SCS.

Our choice of particular miRNAs was based on database research (PubMed). The analyzed miRNAs were considered based on the data regarding their potential relationship with the development of atherosclerosis and their possible prognostic value. Additionally, we included miRNAs that were discriminating biomarkers for the collateral pathway in patients with acute cerebral infarction (Sun et al., 2023). Studies have shown that miR-126, miR-210, and miR-132 can promote angiogenesis, whereas miR-92, miR-15, and miR-16 can inhibit angiogenesis. These miRNAs are related to inflammation (Yin et al., 2012), angiogenesis (Nammian et al., 2020), endothelial and vascular smooth muscle cell differentiation (Martinez-Arroyo et al., 2023), and other processes. Moreover, miR-126 (Liu et al., 2018a) participates in endothelial cell differentiation and angiogenesis through the vascular endothelial growth factor signaling pathway. miR-126, an endothelium-specific miRNA, plays significant roles in physiological angiogenesis (van Solingen et al., 2015). In embryonic vasculogenesis, miR-126 is involved in induction of angiogenic signaling, supports differentiation of embryonic stem cells to endothelial progenitors cells (EPCs) and ECs, and promotes ECs maturation. In a case of vessel injury and/or hypoxia, miR-126 upregulation activates EPCs and ECs and contributes to vascular healing and neovessel formation (Chistiakov et al., 2016). Targeted deletion of miR-126 in mice causes loss of vascular integrity and defects in ECs proliferation, migration, and angiogenesis, which in turn leads to vascular leakage, hemorrhage and partial embryonic death. Mechanistically, miR-126 enhances the proangiogenic effects of VEGF and FGF and promotes angiogenesis by repressing the expression of sprout-related protein 1 (Spred-1), a negative regulator of VEGF signaling (Wang et al., 2008). Similar results are obtained knocking out miR-126 in zebrafish (Fish et al., 2008). Moreover, miR-126 promotes angiogenesis through activation of ERK1/2 and Akt (Shi et al., 2013). Previous studies demonstrated that the expression of miR-126 varies among patients with different types of stroke and higher miR-126 expression are associated with favorable prognosis in patients with acute stroke (Qi et al., 2020). miR-210 [7] is a mediator of hypoxia-inducible factor-1–mediated angiogenesis under hypoxic conditions. It is considered a regulator of vascular smooth muscle cells and endothelial cell biology, contributing to endothelial dysfunction and vascular remodeling and leading to neointimal hyperplasia after vascular injury. miR-210 inhibition greatly decreases the ability of HUVEC to form capillary-like structures in cells exposed to hypoxia and VEGF-driven cell migration. Luciferase reporter assays showed that Ephrin-A3 is a direct target of miR-210. Hypoxia induces downregulation of Ephrin-A3 levels, and inhibition of miR-210 completely blocks the decrease of Ephrin-A3. What’s more, Ephrin-A3downregulation is required for miR-210-mediated stimulation of capillary-like formation and ECs chemotaxis in response to VEGF (Fasanaro et al., 2008). Zeng et al. found correlation of blood miR-210 with clinical findings in acute ischemic stroke involving 112 patients and 60 controls, measured within 3, 7 and 14 days after stroke, using a quantitative PCR technique. MiR-210 level in stroke patients with good outcome and controls was significantly higher in comparison with patients having a poor outcome, suggesting the use of blood miR-210 as a potential biomarker in humans for the prognosis in acute cerebral ischemia (Zeng et al., 2011). miR-132 participates in the migration and proliferation of vascular smooth muscle cells. Exosomes loaded with miR-132 markedly increased tube formation of ECs. subcutaneous injection of HUVECs pretreated with miR-132 exosomes in nude mice dramatically improved their angiogenesis capacity *in vivo*. In addition, transplantation of miR-132 exosomes in the ischemic hearts of mice markedly increased the neovascularization in the peri-infarct zone and improved cardiac functions (Ma et al., 2018). miR-132 promoted endothelial angiogenesis through modulation of the RASA1/VEGF-A signaling pathway and methyl-CpG-binding protein 2 (Anand et al., 2010; Katare et al., 2011). Recently, specific expression patterns of serum miR-132 have been documented associated with various cardiovascular diseases (Xu et al., 2021). Li et al. (2019) evaluated the dynamic changes in plasma levels of miRNAs and cardiac troponin I (cTnI) of 35 acute myocardial infarction (AMI) patients and 55 matched controls, and found that the circulating level of miR-132-5p was maintained at a low level during the early phase of AMI and negatively correlated with cTnI. Liu et al. (2018) found that the plasma levels of miR-132 in heart failure patients with left ventricular ejection fraction less than 45% (*n* = 65) were downregulated compared with healthy controls (*n* = 62). Zeller et al. identified significantly lower miR-132, in unstable angina pectoris (UAP) patients (*n* = 10) than in non-coronary chest pain patients (*n* = 10) and healthy controls (*n* = 10) (Zeller et al., 2014). The above-mentioned studies suggest that a reduction in plasma miR-132 levels, may have a diagnostic value for patients with various cardiovascular diseases. miR-92a inhibits the proliferation of vascular endothelial cells by interfering with the binding and oxidation of low-density lipoproteins (Rani and Sengar, 2022). miR-92a is an endogenous repressor of the angiogenic program in ECs. Moreover, miR-92a inhibition enhances capillary regeneration of ischemic tissue both in mice model of hindlimb ischemia and following MI. Indeed, miR-92a inhibition significantly improved LV systolic and diastolic function in infarcted hearts, reduced the infarct size, suppressed the number of apoptotic cells and significantly augmented the number of vessels, particularly in the infarct border zone. A crucial target of the antiangiogenic action of miR-92a is Integrin-α5, and its downregulation in ECs is able to counteract most of the functions of miR-92a (Bonauer et al., 2009). Deng et al. found that miR-92 was significantly downregulated in arteriosclerosis obliterans (ASO) patients post-surgery, whereas, miR-92 is one of the most upregulated miRNAs during vascular restenosis. Furthermore, KLF4 was validated as a direct target of miR-92, indicating that miR-92 may exert its effects on vascular smooth muscle cells (VSMCs) proliferation, migration, and cell cycle by interaction with KLF4. Accordingly, miR-92 may have a pivotal role in vascular injury and arterial restenosis post-surgery (Deng et al., 2019). The miR-15/16 family restricts the survival and migration of proangiogenic cells and inhibits the formation of new blood vessels (Çakmak and Demir, 2020). MiR-15a and miR-16 are 2 highly conserved miRNAs that are located in a cluster 250-bp apart on chromosome 13q14 in humans and miR-15a/16 cluster was the first identified miRNA group associated with human carcinogenesis. Recently, dysregulation of plasma miR-15a/16 levels has been described in patients with stroke. Wu et al. identified that elevated serum expression of miR-15a and miR-16 was strongly associated with acute ischemic stroke (AIS) and that the combination of these microRNAs may be a promising serum biomarker for AIS (Wu et al., 2015).

After adjusting for age, gender, comorbidities, drinking history and the degree of SCS, logistic regression analysis showed that miR-126-3p had the greatest positive correlation with CCC establishment. In both overall and stratified comparisons, a significant difference in miR-126-3p expression levels was observed between patients with and without CCC (*p* < 0.01). In addition, the ROC curve indicated that miR-126-3p has a strong predictive power for determining CCC status. This suggests that miR-126 can be used as a biomarker for the diagnosis and prognosis of CCC following SCS and is an effective target for drug therapy.

In vascular endothelial cells, miR-126 is uniquely expressed and involved in regulating angiogenesis and vascular integrity (Uniken Venema et al., 2022). It can promote vascular remodeling and reduce fibrosis in multiple organs. It is beneficial for the treatment of atherosclerosis and restenosis (Hui et al., 2020). miR-126 expression in the circulatory system is an indicator of systemic inflammation and angiogenesis (Xu et al., 2021). Patients with ischemic stroke and acute myocardial infarction have significantly reduced blood miR-126 levels. miR-126 upregulation can increase the proliferation and angiogenesis of endothelial cells, promote the formation of new blood vessels in the heart, and improve heart function. In animal models of pulmonary hypertension, upregulation of miR-126 expression by intravenous injection improved cardiac function (Yang et al., 2017). In another experiment (Liu et al., 2018a), *miR-126* was knocked out in mouse endothelial cells, and the distal middle cerebral artery was occluded. After 28 days, vascular endothelial macrophage infiltration and increased oxidative stress were observed in the mice.

This study had certain limitations. First, we only collected blood at a single time point, and the metabolic levels of these miRNAs in the blood were not stable or constant. Future studies should evaluate the association between these miRNAs and collateral circulation via dynamic monitoring. Second, owing to the study design, biomarker measurements were performed after the development of CCC in the SCS group. Therefore, the predictive value of the biomarkers could not be evaluated. Third, the small sample size in our study limited the statistical power; hence, we could not fully assess the association of plasma miRNAs with CCC status.

This study demonstrates that elevated levels of miR-126–3p, miR-132-3p, and miR-210-3p, along with decreased levels of miR-92a-3p, are associated with the development of CCC. These four miRNAs also exhibit promising predictive potential. Notably, miR-126-3p may serve as a potential biomarker for CCC following SCS. Further research is required to validate the clinical relevance of miR-126-3p as a therapeutic target.

## Data Availability

The original contributions presented in the study are included in the article/supplementary material, further inquiries can be directed to the corresponding author.
